# Association Analysis of COQ2 Variant in Dementia and Essential Tremor

**DOI:** 10.1155/2015/926280

**Published:** 2015-04-22

**Authors:** Yin Xia Chao, Ebonne Yu Lin Ng, Huihua Li, Kandiah Nagaendran, Yuen Yih, Mei Sian Chong, Kumar M. Prakash, Louis Tan, Wing Lok Au, Yi Zhao, Zhi Dong Zhou, Murni Tio, Ratnagopal Pavanni, Eng King Tan

**Affiliations:** ^1^Department of Neurology, Singapore General Hospital, Singapore 169608; ^2^National Neuroscience Institute, Singapore 308433; ^3^Duke-NUS Graduate Medical School, Singapore 169857; ^4^Health Assessment Centre, Singapore General Hospital, Singapore 169608; ^5^Health Services Research, Singapore General Hospital, Singapore 169608; ^6^Institute of Geriatrics and Active Ageing, Tan Tock Seng Hospital, Singapore 308433; ^7^Department of Clinical Research, Singapore General Hospital, Singapore 169608

## Abstract

*Objective*. COQ2 mutations have been reported in Japanese multiple system atrophy (MSA) patients. We examined the role of COQ2 in patients with dementia and essential tremor (ET), two common neurodegenerative conditions. *Materials & Methods*. A total of 2064 subjects, including 560 patients with dementia, 466 patients with ET, and 1038 healthy controls, were included. Genotyping for the COQ2 V393A (T>C) was carried out. Odds ratio (OR) adjusted by age and gender, together with 95% confidence interval (CI), was reported by means of logistic regression. *Results*. The frequency of the polymorphic variant V393A heterozygous (T/C) was 2.7% in dementia, 1.1% in ET, and 2.5% in controls (OR = 0.70, 95% confidence interval is 0.29–1.72 for dementia, and OR = 0.47, 95% confidence interval is 0.17–1.31, *p* = 0.1217 for ET). There was no significant association between V393A variant with dementia and ET. *Conclusion*. There was no significant association between V393A variant with dementia and ET. COQ2 gene is unlikely to play a significant role in patients with dementia or ET in our population.

## 1. Introduction

Coenzyme Q10 (COQ10), also known as ubiquinone, ubidecarenone, and coenzyme Q, is an oxidative phosphorylation enzyme and plays a unique role in the electron transport chain (ETC) in mitochondria. COQ10 functions as an electron carrier from enzyme complex I and enzymes complex II to complex III in the process of ATP production. Vitamin K2 coperforms this role with COQ10 [1]. COQ10 functions in every cell of the body to synthesize energy and may be an important factor in neurodegeneration [2]. The gene COQ2 is critical in COQ10 biosynthesis [3].

Mutations/variants of COQ2 have been found to modulate the risk of familial and sporadic multiple system atrophy (MSA) in Japan, supported by impaired COQ2 activities in affected subjects. A homozygous mutation (M78V-V393A/M78V-V393A) and compound heterozygous mutations (R337X/V393A) have been identified in the coenzyme Q2 4-hydroxybenzoate polyprenyl transferase (COQ2) gene in two familial MSA cases by using a combination method of whole-genome sequencing and linkage analysis. The common variant Val393Ala and multiple rare variants in COQ2 are associated with increased risk of MSA in familial and sporadic Japanese patients. However, this association was not observed in 223 European and 172 North American MSA patients (the allele frequency of V393A in patients is 0% in these cohorts) [4]. Whether COQ2 mutation is ethnic specific or disease specific is yet to be investigated.

Since COQ2 is vital for redox balance in neurons and the role of COQ2 variants is unknown in other neurodegenerative diseases such as dementia and essential tremor, we conducted an association analysis of COQ2 variants in a cohort of dementia and essential tremor (ET) patients.

## 2. Materials and Methods

### 2.1. Subjects

The study subjects were recruited from two tertiary centres. Clinical information, including sex, age, age of onset, and family history, was also collected. The dementia group included 347 AD and 213 mixed dementia (AD plus vascular dementia) patients who satisfied established clinical criteria. The dementia syndrome was diagnosed using the* Diagnostic and Statistical Manual, 4th Edition* (DSM-IV) criteria [9] and the aetiological diagnoses followed the criteria of the National Institute of Neurological and Communicative Diseases and Stroke/Alzheimer's Disease and Related Disorders Association (NINCDS-ADRDA) for Alzheimer's disease (AD) [10]. The diagnosis of ET was based on the Consensus Statement of the Movement Disorders Society in 1998 [11]. Healthy controls were recruited from community health screening centre. All of the control subjects were examined by the authors to rule out any clinical evidence of neurodegenerative disorders. Written and signed informed consent forms were obtained from all participants. For cognitively impaired subjects who were not mentally able to give consent, the next of kin or legally authorised representative consented on the behalf of the subjects. The study was approved by the SingHealth Centralised Institutional Review Board (CIRB) and National Healthcare Group Domain Specific Review Board (DSRB).

### 2.2. DNA Preparation, Polymerase Chain Reaction (PCR), and DNA Sequencing

Genomic DNA was extracted from venous blood drawn using QIAGEN QIAamp Blood Kit. SNPs of COQ2 were determined using a Real Time 7500 PCR platform (Life Technologies) using the primers (forward: 5′-TTATTTGTTATGACCCAAAGTCC-3′; reverse: 5′-GCTCTAAATCTTCATCTTCAGGTTC-3′) for V393A. Genotyping for the COQ2 polymorphic variant V393A (T>C) was carried out using the allelic discrimination method followed by sequencing for representative samples.

### 2.3. Statistical Analysis

Median together with range was reported for continuous variables, while frequency together with proportion was reported for categorical variables. Mann-Whitney *U* test was performed to compare continuous variables between cases and controls, while Fisher's exact test was carried out to compare categorical data between these groups. The association between SNPs and dementia/ET was evaluated by means of logistic regression. Odds ratio (OR) was adjusted by age, gender, and race considering the difference between cases and controls. All analysis was done using R 3.0.2 (http://www.r-project.org/) with 2-sided significance level of 0.05. Power was calculated with Genetic Power Calculator (http://pngu.mgh.harvard.edu/~purcell/gpc/).

## 3. Results

### 3.1. Clinical Data

A total of 2064 subjects, including 560 patients with dementia, 466 patients with ET, and 1038 healthy controls, were included. The mean and median age of dementia patients were 73 and 75 years (ranging from 40 years to 93 years), 51 and 55 years for ET (ranging from 15 years to 86 years), and 53 and 53 years for controls (ranging from 24 years to 90 years). There were 258 males and 302 females in dementia group and 261 males and 205 females for ET group and 570 males and 468 females for the control group. Their demographic data are summarized in Table 1.

### 3.2. Genetic Analysis

Our study has 90% power to detect the effect size difference reported in MSA patients at alpha = 0.05. Fifteen dementia patients (2.7%), 5 ET patients (1.1%), and 26 controls (2.5%) were found to carry the heterozygous variants. Two dementia patients and 1 control were found to carry the homozygous mutation while there was no homozygous mutation in essential tremor patients. No significant differences existed in the genotype frequency distribution (OR = 0.70, 95% confidence interval is 0.29–1.72 for dementia with T/C heterozygous, OR = 0.51, 95% confidence interval is 0.01–30.40 for dementia with C/C homozygous, Table 2, and OR = 0.47, 95% confidence interval is 0.17–1.31; *p* = 0.1217 for ET; Table 3). Odds ratios were adjusted by gender and age in a multivariate analysis.

## 4. Discussion

The COQ2 gene is located at chromosome 4q21.23 and includes seven exons. The protein COQ2 is widely expressed in all tissues, especially at high level in the skeletal muscle, adrenal glands, and heart [12]. COQ2 located at the mitochondrion membrane catalyzes the prenylation of para-hydroxybenzoate, which has a main function in the biosynthesis of ubiquinone (coenzyme Q) [12, 13]. COQ is a redox carrier in the mitochondrial respiratory chain, and the only lipid-soluble antioxidant endogenously synthesized in both unicellular and multicellular organisms [14]. Tissue levels of COQ are associated with different disorders. Neurodegenerative disorders, cardiovascular diseases, cancer, diabetes mellitus, and especially aging and Alzheimer's disease exhibit altered levels of COQ, indicating their likely crucial role in the pathogenesis and cellular mechanisms of these ailments [15]. Previous studies have reported that mutation in the COQ2 caused primary coenzyme Q10 (COQ10) deficiency, a disorder mainly involving the nervous system [16]. Functionally impaired variants of COQ2 have also been associated with increased risk of MSA in Japanese population [4]. While some reports indicate that COQ2 mutation was not associated with Parkinson's disease and Amyotrophic Lateral Sclerosis (ALS) (Table 4) [4–8], the association between COQ2 mutation and dementia and ET is not clear. Given the fact that these disorders share some common pathological changes such as the Lewy body formation and synucleinopathy, we hypothesize that COQ2 mutation may also be involved in the pathogenesis of these diseases.

To the best of our knowledge, this is the first study to investigate the association between the Val393Ala of the COQ2 gene and dementia and ET. Utilizing a case control methodology, we demonstrated that the COQ2 polymorphic variant V393A (T>C) is present in non-Japanese population. However, there was no association between this variant and dementia or ET patients. Future studies in different ethnic populations are warranted to further validate our findings.

## 5. Conclusion

There was no significant association between V393A variant with dementia and ET. COQ2 gene is unlikely to play a significant role in dementia and essential tremor in our population.

## Figures and Tables

**Table 1:** Demographics of dementia, ET, and controls.

	Controls	Dementia	ET
Age	53 (24, 90)	74 (40, 92)	40 (2, 85)
Gender			
Male	570 (54.9%)	258 (46.1%)	261 (56.0%)
Female	468 (45.1%)	302 (53.9%)	205 (44.0%)

**Table 2:** Logistic regression analysis showing no association between individual SNP and dementia.

Genotype	Controls	Cases	OR
T/T	1011	543	Reference
T/C	26	15	0.70 (0.29, 1.72)
C/C	1	2	0.51 (0.01, 30.40)

OR: odds ratio adjusted by gender and age.

**Table 3:** Logistic regression analysis showing no association between individual SNP and ET.

Genotype	Controls	Cases	OR
T/T	1011	461	Reference
T/C	26	5	0.48 (0.17, 1.36)
C/C	1	0	—
Dominant model			0.47 (0.17, 1.31)
*p* value of dominant model			0.1217

OR: odds ratio adjusted by gender and age.

**Table 4:** Summary of reported COQ2 variant V393A screening in neurodegenerative disorders.

	MSA	PD	ALS
Mitsui et al. [4]	Positive (OR = 3.05)	Negative	
Lin et al. [5, 6]	Positive (OR = 3.10)	Negative	
Chen et al. [7]	Negative		
Yang et al. [8]			Negative
Lin et al. [5, 6]		Negative	

## References

[1] Bhalerao S., Clandinin T. R. (2012). Cell biology. Vitamin K2 takes charge. *Science*.

[2] Salama M., Yuan T. F., Machado S. (2013). Co-Enzyme Q10 to treat neurological disorders: basic mechanisms, clinical outcomes, and future research direction. *CNS and Neurological Disorders—Drug Targets*.

[3] Quinzii C., Naini A., Salviati L. (2006). A mutation in para-hydroxybenzoate-polyprenyl transferase (COQ2) causes primary coenzyme Q_10_ deficiency. *The American Journal of Human Genetics*.

[4] Mitsui J., Matsukawa T., Ishiura H. (2013). Mutations in *COQ2* in familial and sporadic multiple-system atrophy. *The New England Journal of Medicine*.

[5] Lin C. H., Tan E. K., Yang C. C., Yi Z., Wu R. M. (2015). *COQ2* gene variants associate with cerebellar subtype of multiple system atrophy in Chinese. *Movement Disorders*.

[6] Lin C. H., Lin H. I., Chen M. L., Wu R. M. (2015). COQ2 p.V393A variant, rs148156462, is not associated with Parkinson's disease in a Taiwanese population. *Neurobiology of Aging*.

[7] Chen YP., Zhao B., Cao B. (2015). Mutation scanning of the COQ2 gene in ethnic Chinese patients with multiple-system atrophy. *Neurobiology of Aging*.

[8] Yang X., Xi J., An R. (2015). Lack of evidence for an association between the V393A variant of COQ2 and amyotrophic lateral sclerosis in a Han Chinese population. *Neurological Sciences*.

[9] American Psychiatric Association (1994). *Diagnostic and Statistical Manual of Mental Disorders: DSM-IV*.

[10] McKhann G., Drachman D., Folstein M. (1984). Clinical diagnosis of Alzheimer's disease: report of the NINCDS-ADRDA work group under the auspices of Department of Health and Human Services Task Force on Alzheimer's disease. *Neurology*.

[11] Deuschl G., Bain P., Brin M. (1998). Consensus statement of the movement disorder society on tremor. Ad Hoc scientific committee. *Movement Disorders*.

[12] Forsgren M., Attersand A., Lake S. (2004). Isolation and functional expression of human COQ2, a gene encoding a polyprenyl transferase involved in the synthesis of CoQ. *Biochemical Journal*.

[13] Jakobs B. S., van den Heuvel L. P., Smeets R. J. P. (2013). A novel mutation in COQ2 leading to fatal infantile multisystem disease. *Journal of the Neurological Sciences*.

[14] Turunen M., Olsson J., Dallner G. (2004). Metabolism and function of coenzyme Q. *Biochimica et Biophysica Acta—Biomembranes*.

[15] Dhanasekaran M., Ren J. (2005). The emerging role of coenzyme Q-10 in aging, neurodegeneration, cardiovascular disease, cancer and diabetes mellitus. *Current Neurovascular Research*.

[16] Desbats M. A., Lunardi G., Doimo M., Trevisson E., Salviati L. (2015). Genetic bases and clinical manifestations of coenzyme Q_10_ (CoQ_10_) deficiency. *Journal of Inherited Metabolic Disease*.

